# Advances in Diagnosis and Management of Lateral Ankle Instability: A Review of Current Literature

**DOI:** 10.5435/JAAOSGlobal-D-23-00251

**Published:** 2023-12-26

**Authors:** Amiethab Aiyer, Sudarsan Murali, Anish R. Kadakia

**Affiliations:** From the Department of Orthopaedic Surgery, Johns Hopkins University, Baltimore, MD (Aiyer and Murali), Department of Orthopaedic Surgery, Northwestern University, Evanston, IL (Kadakia).

## Abstract

Lateral ankle sprains and instability are an increasingly identified pain point for patients, accounting for 20 to 25% of musculoskeletal injuries. Lateral ankle injuries are especially concerning given the propensity for patients to develop chronic lateral ankle instability and for the high risk of reinjury on an unstable ankle. With the complex articulation of the tibiofibular syndesmosis, subtalar, and talocrural joints, pinpointing ankle dysfunction remains difficult. Multiple reviews have evaluated management and diagnosis of lateral ankle instability, but with newer treatment options available, a more comprehensive assessment of the current literature was conducted. Although multiple surgical options exist, many nonsurgical functional options have also been developed for patients that may help patients prevent the development of chronic lateral ankle instability. In recent times, many new options have come up, including in-office needle arthroscopy and continual advancements in diagnosis and our understanding of this difficult topic. Multiple reviews have evaluated the management and diagnosis of lateral ankle instability, but with newer treatment options available, a more comprehensive assessment of the current literature was conducted. Given this, this review will help to highlight new diagnostic and nonsurgical therapeutic options for the management of lateral ankle instability.

Acute ankle injuries continue to be a common issue treated by orthopaedic surgeons and sports medicine physicians with many diagnostic and treatment options. Among ankle injuries, those to the lateral ankle are more prevalent and compose the cohort of patients who experience acute lateral ankle instability (ALAI).^1^ Of those who experience acute injuries of the lateral ankle, many are amenable to conservative approaches (rehabilitative and minimally invasive therapies), but a subgroup of up to 40% continue to develop into chronic lateral ankle instability (CLAI), highlighting the importance of optimizing interventions to help decrease this cohort.^2^ In addition, many of these patients with ALAI or CLAI are predisposed to future injuries, making it important for physicians to ensure patients not only recover but also bolster their lateral ankle complex to help prevent future recurrence.^3^ This article will review the lateral ankle complex, diagnosis, minimally invasive treatments, and preventive options for patients with lateral ankle instability.

## Anatomy

The subtalar, talocrural, and tibiofibular joints provide most of the articulation and movement within the ankle. Owing to the complexity of the ankle, most functional motion stems from the unified action of one or more of these joints to create the traditional motions of the ankle. Alterations of the various articulation points lead to significant derangements of the ankle. In particular, the lateral ankle lies at the intersection of the tibia, fibula, talus, and calcaneus, unified with a few key ligaments that provide lateral ankle stability.^4^ The most commonly injured ligament being the anterior talofibular ligament (ATFL), and this ligament provides resistance to inversion and anterolateral translation to the ankle mortise.^5^ In some studies, the ATFL shows lower load capacitance and higher failure rates under stress compared with other lateral ligaments, such as the calcaneofibular ligament (CFL) and posterior talofibular ligament.^6^ By contrast, the CFL originates off the distal tip of the fibular near the ATFL and inserts onto the calcaneus providing resistance to inversion stresses. The CFL has been shown to hold a larger maximum load suggesting its increased strength and potentially explaining the lower injury rates.^7^ Finally, the posterior tibiofibular ligament and the tibiotalar ligament help provide resistance from inversion and anterior displacement, respectively.^8^

When considering the anatomy of the lateral ankle, it is also clinically important to consider the dynamic stability that the myotendinous units traversing the lateral ankle have on the protection of the osseous restraints and ligamentous tissue. The peroneal longus and brevis cross the lateral ankle and function to support pronation and eversion while protecting against inversion injuries of the lateral ankle ligaments.^9^ In particular, some studies show that patients with ankle sprains more frequently have weakness of the peroneal muscles contributing to their ankle instability and highlighting the importance of the muscular stabilization of the lateral ankle.^10,11^ Additional support is also provided by the tibialis anterior and its protection against posterior translation and excessive dorsiflexion, while the tibialis posterior helps prevent anterior translation and help support against flexion and extension moment arms.^12-14^

## Presentation and Diagnosis

All workup of lateral ankle instability should include a thorough history of the current condition and a history of prior ankle instability and injuries. Many additional risk factors have also been identified including intrinsic risk factors, such as younger age and body mass index.^15^ Particularly in athletes, a detailed history of muscle strength and postural work has been shown to be correlated with lateral ankle instability, showing that those that do not engage in these types of activities are at higher risk of injury.^15,16^

### Physical Examination

Many different physical examination modalities exist for the ankle, but when looking at reported sensitivity and specificity data, the importance of palpation of the ligaments has shown to play an important role in diagnosis with sensitivities ranging from 78 to 92% in some studies.^17,18^ With minimal superficial overlying tissue, palpation of the ATFL and CFL provides a great starting point for examination of the lateral ankle. For the ATFL, in particular, some common tests include the standard anterior drawer test and the anterolateral drawer, with the literature suggesting superior sensitivity and specificity with the anterolateral drawer in comparison with the anterior drawer.^19^ In the anterior drawer test, the examiner stabilizes the talofibular joint while using the other hand to hold the heel and translate it anteriorly. If this same motion is replicated with the ankle in maximal dorsiflexion, it is called an anterolateral drawer. More recent literature also suggests the use of a reverse anterolateral drawer with greater sensitivity, especially for more chronic tears.^20^ In the reverse anterolateral drawer, the hindfoot is stabilized while a posterior force is applied onto the tibia. The anterior drawer can also help identify pathology of the CFL in conjunction with the ATFL particularly when done with dorsiflexion, but it is not a very sensitive for isolated CFL injuries.^21^ Similarly, talar tilt can also be used to test for the CFL particularly when done in 10° of dorsiflexion to help isolate the CFL, but small differences that may be present are hard to identify on examination.^22^ By contrast, combined ATFL and CFL injuries produce larger degree motion during the test, improving detection of the positive test.^22^ No key physical examination findings have been shown to preclude advanced imaging if there is concern for lateral ankle instability (LAI), but examination of the ligaments for tenderness and laxity does have a high sensitivity that could be used to decrease the clinical suspicion for LAI and therefore further imaging in the right clinical scenario. It is also important to consider hindfoot alignment considerations that could increase stress on the lateral-sided ligaments and increase risk of acute injury.^16^

### Imaging

When evaluating the lateral ankle, three key imaging modalities exist. Classically, stress radiography could help show increased motion along the lateral ankle, but new options, such as ultrasonography stress tests and MRI imaging, particularly T2 series, have also gained traction among clinicians. Looking at the diagnostic efficacy, stress radiography shows sensitivity around 80% for ATFL injuries and around 50% for CFL injuries.^23^ For ultrasonography stress tests, a systematic review showed pooled sensitivities of around 99% for ATFL injuries and 94% for CFL injuries.^23^ Finally, MRI showed the sensitivities around 80% for complete ATFL tears and 95% for the CFL. Given this and the availability of ultrasonography in many office settings, ultrasonography guided stress tests seem to provide a great adjunct to physical examination maneuvers and also provide live feedback during manipulation of the ankle. For ultrasonography stress tests, the imaging can be obtained with the patient's foot resting in a neutral position with 15° to 20° of dorsiflexion. The transducer can be placed parallel to the plantar surface along the ATFL while the ATFL is stressed to visualize laxity. In addition, ultrasonography also offers support for early treatment options including injected biologics or anti-inflammatory agents that radiography and MRI do not provide. Looking at the downsides of each option, however, ultrasonography is a newer skill and has a learning curve associated with it although it is inexpensive and offers dynamic visualization in the hands of the clinician. On the other hand, MRI is expensive and harder to schedule and obtain but is well-described in the literature and provides the input of a radiologists read as well.

### In-Office Arthroscopy

New techniques are now also being introduced in the field of foot and ankle surgery. Among these, in-office needle arthroscopy (IONA) offers a unique option to directly visualize the ankle joint and the supporting structures in real time. Currently, it is used in visualizing syndesmosis injuries and osteochondral lesions of the talus, and expanding to lateral ankle instability is a natural next step. Given the limited research surrounding IONA, no firm consensus exists regarding the sensitivity, specificity, and indications for use. Despite this, preliminary case series do suggest that IONA may play a role in advancing the options available to clinicians in the diagnosis and management of LAI. In a comparative case series, it was demonstrated that the needle arthroscope provided good visualization of the anterior compartment of the ankle and syndesmosis, but difficult views of the medial gutter and lateral gutter when using a standard anterolateral portal.^24^ Owing to the scope availability, only a 0° scope is available for IONA compared with 30 and 70° scopes for traditional arthroscopy. If using, it as an alternative to MRI, and this might provide a potential avenue for immediate diagnosis as discussed by authors in one study.^24^ Despite its limitations, surgeons are beginning to expand its use from a purely diagnostic tool to a therapeutic modality by repairing the ATFL with this technology in the operating room.^25^

## Management

### Nonsurgical

Many principles in nonsurgical treatment of lateral ankle instability exist, but the primary goal of all therapies is to improve the function of the dynamic stabilizers of the ankle and is always indicated for purely ligamentous injuries before surgery. Aggressive functional rehabilitation with range of motion and advancing weight-bearing status should be delayed in cases that present with bony abnormalities, such as lateral process fractures or anterior process fractures. In addition, suspected syndesmotic injuries or avulsion fractures may respond better to a more conservative treatment with prolonged use of a LAI walker as opposed to the rapid functional rehab that is used for a lateral ankle sprain.

When conservative therapy is identified as an option, it is important to identify signs of treatment failure based on patient-specific factors and goals of care to identify the need to advance treatment to surgical options. No firm end points have been established to label the failure of conservative management, although traditionally 3 to 6 months without improvement is widely used.^26,27^ Among the external stabilizing devices used to facilitate this, popular options include ankle orthoses and ankle taping. Although these orthoses do not directly rectify the issue, they do prevent the negative consequences of ankle laxity by providing the external support needed to correct ankle motion as shown by some studies, especially when custom-molded orthoses are used.^28,29^ In fact, when evaluating inversion angles and velocity at the ankle, there was a significant reduction in both parameters with the use of bracing treatment.^30^

Physical therapy traditionally focuses on strengthening of the peroneal muscles to prevent inversion injuries and closed chain activity to help optimize foot strike and mechanics.^26^ In addition to physical therapy on the muscles surrounding the ankle, newer literature has also found benefit to strengthening the muscles proximally in the lower extremity as well, including the gluteal musculature. Some studies have found that there is a significantly different gluteus medius activity in patients with lateral ankle instability, suggesting another area to focus on to help create an all-encompassing plan treatment plan for ankle instability.^31,32^

In addition to the gluteus muscles, another area of interest includes core strengthening. One study showed that those who engaged in core stability work helped improve control of the ankle and serves as a potential avenue for managing patients with lateral ankle instability.^33,34^ In addition, these findings also point to an interesting avenue for not only treatment and management but also potentially prevention of these injuries.^35^ Particularly in athletic cohorts where the incidence is the highest, early intervention to work on dynamic strengthening and kinematics may be an important avenue for preventing new ALAIs and progression to CLAI.^3^ Historically, nonsurgical management has focused on direct ankle stabilization and rehab, but newer literature emphasizes the holistic approach to rehabilitation with increased emphasis on core and gluteal stabilization to improve lateral ankle instability.

Among the nonsurgical options, the role of biologic therapeutics has also been on the rise in the field of orthopaedics, although the evidence to support their use is limited. As the body of literature grows in this area, more clarity may be achieved, but for now, these remain an expensive option with limited support for their use. The use of platelet-rich plasma (PRP) therapy has grown significantly in many areas of orthopaedics, and research in the setting of lateral ankle instability does show that there may be a role for this therapy for management. In one study, PRP injections showed similar outcomes compared with rigid immobilization at the 24-week mark and less pain at the 8-week mark.^36^ Another study also corroborated this finding of early pain relief, but at the 6-month mark, there was no difference between those who had received PRP and those who had not.^37^ Other biologics under investigation include bone marrow concentrate, stem cells, and hyaluronic acid, but no conclusive literature has been published, although the use of these as augments to surgical and nonsurgical options does seem to be promising.^38^ In summary, first-line therapy remains taping and bracing treatment with physical therapy of the ankle, glute, and core for stability with expected progress and return to activity in 6 to 12 weeks. If the patient is not progressing further, imaging and workup for surgical therapy are indicated in most patients based on their goals of care.

### Operative

Surgical options for lateral ankle instability are a well-investigated and discussed topic, but most repairs consist of ligamentous reconstruction of the ATFL and/or the CFL in an anatomic or nonanatomic fashion to preserve the ankle range of motion. The most classic anatomic surgical option includes the Brostrom technique and its numerous modifications, including newer ligamentous or suture tape augments for stability.^27^ In one comparative study, these anatomic techniques showed to be superior to nonanatomic options that were tested.^39^ Despite this and the growing body of literature, however, there is no consistent evidence for one technique over the other, but in general, studies support the utility of the anatomic techniques for their ability to allow earlier and more aggressive rehabilitation.^27,40^

## Summary

In summary, lateral ankle instability is a complex topic of growing interest within the field of orthopaedic surgery. In the diagnostic process, ultrasonography has been shown to be more sensitive lateral ankle evaluation in comparison with the traditional radiographic stress, although it is limited by the learning curve surrounding its use. Nonsurgical management remains the first-line therapy for ligamentous injury, but newer literature emphasizes the importance of incorporating gluteal and core strengthening in physical therapy regimens. In addition, biological agents are continuing to gain traction and may provide augmentation to nonsurgical management, but the literature on outcomes is still lacking. Once surgery is considered, new techniques, such as IONA, are being investigated for minimally invasive surgical and diagnostic options, but it remains to be seen if the outcomes are equivocal with future research studies. Further interest and research continue to drive the current recommendations and indications for management of CLAI and ALAI, and more preventive options with the knowledge over current physical therapy regimens will likely continue to help address the incidence of CLAI and ALAI.
